# Voices in Motion: How Social Singing Can Facilitate Psychological Health for Persons With Dementia and Caregivers

**DOI:** 10.1093/geroni/igab046.495

**Published:** 2021-12-17

**Authors:** Stuart MacDonald, Debra Sheets, Theresa Allison

## Abstract

Dementia is a global public health priority that exerts significant impact on individuals, families, healthcare systems, and society. Worldwide, over 35 million individuals are estimated to have some subtype of dementia – a projection expected to triple by 2050. Despite progress, a cure for dementia remains elusive, and approved pharmacotherapies are selectively effective for but a limited duration. Increasingly, arts-based interventions for persons with dementia (PwD) and their caregivers are being recognized as inexpensive, non-invasive, and non-pharmacological lifestyle interventions with the potential to improve psychological function as well as reduce healthcare costs. This symposium overviews the Voices in Motion (ViM) study – a sociocognitive intervention exploring the impact of participation in an intergenerational choir on psychological, social, and cognitive function for PwD and their caregivers (n=32 dyads). PwD, caregivers, and local high school students sang in a professionally-conducted choir for as many as three seasons (each ~12 weeks long), spanning up to 18 months of follow-up. Employing an intensive repeated measures design, psychosocial, physiological, and cognitive function was measured every four to six weeks (up to 9 total assessments). The symposium papers provide a representative overview of the broad impact that this novel, non-pharmacological lifestyle intervention can offer vis-à-vis mitigating dementia symptoms and facilitating the psychological health of caregivers. Choir participation has important and significant impacts on psychosocial well-being and quality of life. Discussion focuses on policy implications and the need for community-based programs that reflect a social model for dementia and support living well through engaging and meaningful activities.

